# Psychotherapeutic Consultation Services in the Workplace: A Longitudinal Analysis of Treatments and Sick Leave Using Health Insurance Data

**DOI:** 10.3389/fpsyt.2022.838823

**Published:** 2022-03-24

**Authors:** Melanie Gantner, Marc Nicolas Jarzcok, Jürgen Schneider, Stefan Brandner, Harald Gündel, Jörn von Wietersheim

**Affiliations:** ^1^Department of Psychosomatic Medicine and Psychotherapy, University Medical Centre, Ulm, Germany; ^2^Wieland BKK, Ulm, Germany

**Keywords:** collaborative care in the workplace, sick leave, health services research, workplace intervention, occupational health service, workplace mental health

## Abstract

**Background:**

Psychotherapeutic consultation services in the workplace (PSIW) have been developed to provide collaborative mental health care for employees. The aim of this study was to analyze participant characteristics, the role of PSIW in treatment courses, and the development of sick leave before and after PSIW start.

**Methods:**

Routine data from PSIW and health insurance of 155 participants were analyzed descriptively and by means of a multilevel negative binomial regression.

**Results:**

Eighty-four percent of users were male, and 72% were diagnosed with a mental disorder. The number of PSIW consultations varied from 1 to 13 (mean = 4). For 34% of participants, PSIW sessions were sufficient, 33% received a recommendation for outpatient psychotherapy, and 20% for inpatient mental health treatment. While recommendations for inpatient treatment displayed a high adherence rate (74%), recommendations for outpatient treatment were followed by 37%. Compared with the period of a half-year before PSIW, sick-leave days were reduced from the period of the second half-year after PSIW start and in the subsequent observed half-year periods. Trajectories of sick leave by subgroups showed differences.

**Conclusions:**

PSIW is a flexible care offer, and results indicate a possible effect of PSIW on sick leave. In future studies, control group designs and inclusion of further variables are needed.

## Introduction

An estimated 20% of the working-age population in industrial countries suffer from mental illness at a given time, mostly from common mental disorders such as depression and anxiety (1). For the affected individual, this usually implies a heavy burden of distress, including the impairment of work ability as an essential source of psychosocial well-being (2–4). Mental disorders correlate with reduced productivity in terms of presenteeism and increased sick leave (5–8). In total, direct and indirect costs of mental illness in European countries are estimated at ~3.5% of the gross domestic product (1). Despite these consequences, treatment is often substantially delayed, or there is no treatment at all (9). Even in high-income countries, only one in five individuals suffering from, for example, major depressive disorder (MDD) received minimally adequate treatment within the last 12 months (10). As the COVID-19 pandemic seems to have negative effects on mental health, the issue of providing adequate prevention and treatment becomes even more important (11, 12).

In Germany, mental disorders have been the cause of rising numbers of sick-leave days, accounting for 13–17% of all sick-leave days over the last 10 years (13–16). They have also become the leading cause of disability benefits (17). Specialist mental health care in Germany is provided by private practitioners (specialized physicians or psychotherapists) or by psychiatric/psychotherapeutic hospitals. Costs are usually covered by health insurances, and nearly 100% of the population have such an insurance (18). Nonetheless, approximately only 20% of the people diagnosed with a mental disorder have made use of the health care offers within the preceding year (19). One critical factor for this treatment gap seems to be the limited accessibility and substantial waiting time for outpatient psychotherapy (20, 21). Besides, individual factors such as shame, fear of stigmatization, or limited knowledge about causes and treatment options for mental disorders can lead to delayed or no treatment at all (22–24). This seems to be even more pronounced in men compared with women (19, 25).

As a consequence, there is need for collaborative care offering early identification and treatments for mental health problems, as well as improved support to return to work (1). Collaborative mental health care in the workplace (CMHW) links company health promotion with regular health care and has the potential to lower the threshold to make use of appropriate care and to facilitate staying at or returning to work (26).

One of the recently developed concepts of CMHW in Germany is the “psychotherapeutic consultation service in the workplace” (PSIW) (27–29). PSIW mainly aims to reach employees with symptoms at a subsyndromal stage, but also early in the course of apparent diseases or when in need of support to return to work (30). Consultations are provided by a medical or psychological psychotherapist of cooperating institutions and take place either in company buildings or in cooperating clinics or practices. Costs are covered either directly by the companies or by the company health insurance funds. Depending on company-specific regulations, either employees are referred to PSIW by company stakeholders such as occupational physicians, social workers, or employee representatives, or employees are free to register without referral. PSIW sessions include several basic elements (31, 32): not only clinical symptoms, functional impairment, and need for further treatment (e.g., regular outpatient or inpatient mental health care) are assessed by therapists. Here, unlike often in standard care, the workplace (as a resource or burden) is explicitly considered. Participants receive information about their symptoms and possible self-help options. Recommendations for further treatments are given, and access to treatment is facilitated by information and motivation. If possible, PSIW also offers a short-term individualized psychotherapeutic intervention that includes work-oriented content when appropriate. This can work either as a stand-alone intervention or to support the participant until the start of other treatments. In addition, staying at or returning to work might be facilitated by assistance from collaborating company physicians or social workers when the participant consents.

Recent reviews and meta-analyses report on mental health interventions in the workplace including a wide range of settings, target groups, interventions, outcomes, and with differing results (33–35). Symptom severity and capacity to work are not necessarily closely related (35). Restoring an employee's capacity to work capacity often follows symptom reduction (36), and interventions on merely the individual level are not always sufficient (37). A meta-analysis for psychological interventions found reduced symptoms and sick leave compared with care as usual with small effect sizes. However, results on the reduction on sick leave were still considered to be very heterogeneous, and more research with consensual measures of sick leave is required (33). Concerning interventions for return to work, meta-analytic results showed no effect of psychological therapies alone but an increased effectiveness when contact with the workplace or multiple components (e.g., psychological interventions and graded return to work) are included (34).

Only few research data exist for CMHW concepts in Germany (38). First research on PSIW shows that participant acceptance and satisfaction are high (32, 39–41). Compared with an outpatient unit of a psychosomatic clinic, participants can be reached at earlier stages of the symptom development (42), and symptoms can be reduced (28, 43). With regard to participant characteristics and pre-treatments and post-treatments, reports differ depending on the sample characteristics (39, 41, 42, 44, 45). First positive results regarding the improvement of work outcomes exist, but they are based on subjective questionnaire data or impaired subgroups (i.e., with MDD or already on sick leave) (28, 44, 46).

We chose to use the advantages of secondary data from a company health insurance fund (47, 48). Health insurance data are not biased by self-selection of patients (i.e., due to severity of sickness or satisfaction with treatment), or any kind of recall bias (49, 50). Furthermore, data are available over long time spans and allow a longitudinal perspective with precise time frames. As such, the analysis of health insurance data offers a useful addition to randomized trials or questionnaire data (51).

In the present study, we aimed to explore participant characteristics and the role of PSIW in the treatment course of employees with mental health issues. Second, we investigated the longitudinal course of sick leave 1 year before and 2 years after first PSIW session. We intended to gain more information about the possible effects of PSIW on sick leave and about the relevance of participant subgroups. Also, indications for more specific research questions and designs should be derived. To our knowledge, this is the first study to use insurance data for the analysis of sick-leave days while including the entire spectrum of PSIW participants. For this purpose, the following research questions were addressed:

What are the characteristics of PSIW participants in terms of preceding treatment, mental illness, and need for treatment? How many PSIW participants use the recommended treatments?

What trajectories of sick-leave days can be observed? Is there a reduction in sick leave following the start of PSIW compared with before PSIW?

## Materials and Methods

### Study Design, Sample, and Data Sources

The present study has a naturalistic longitudinal design using health insurance data combined with data assessed during the PSIW. More detailed descriptions of the materials and methods are available (52).

The final sample consisted of 155 PSIW participants. Of 184 employees of a metal works company in southern Germany who started and finished their PSIW consultations between August 2011 and May 2014, we excluded 26 participants who were not insured by the company health insurance and three people who could not be matched to health insurance data.

PSIW was offered according to the concept described above. For the PSIW of the investigated company, the following specifications were applied: employees were referred to the PSIW by occupational physicians. They received individually scheduled sessions provided by a mental health specialist of the Department of Psychosomatic Medicine and Psychotherapy of Ulm University Medical Centre (psychological or medical psychotherapist). After assessment of symptoms and need for treatment, participants received a short non-standardized psychotherapeutic intervention with a general maximum of 12 PSIW sessions in total when suitable.

To answer our research questions, we used insurance data on medically certified sick leave as well as inpatient and outpatient treatment data, including diagnoses and procedures. These data were linked to the information assessed during the PSIW consultations (dates of consultation, diagnosis, and recommended further treatment). Data were analyzed in an anonymized way according to the German Federal Data Protection Act (BDSG 24.05.2018 §3). The data transfer and analysis procedure were approved beforehand by the commission for data security of Ulm University Medical Centre, Ulm, Germany. The study was also approved by the local ethics committee of Ulm University Medical Centre, Ulm, Germany (Ref No. 53/16).

**Figure 1:**
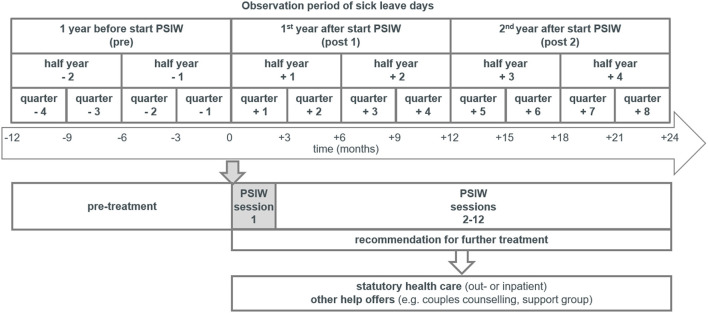
Timeline of psychotherapeutic consultation services in the workplace (PSIW) and observation periods of sick leave.

The observation periods covered 1 year before first PSIW consultation (pre) and 2 years after (post) (Figure 1). Of the included 155, eight subjects were not insured over the entire observation period of 3 years, therefore creating missing data for some variables either in the pre-observation or post-observation period.

In pre–post studies that investigate effects of psychotherapeutic interventions targeting symptom reduction or sick leave, time of treatment is often excluded from the analysis (53–56). As treatment paths of PSIW participants are very heterogeneous and subject of our interest, we wanted to obtain a continuous observation of treatments and sick leave as measured in days. On the basis of clinical experience and results of studies on outpatient psychotherapy in Germany (53, 57–59), we assumed that in the first year after PSIW treatments (and symptom reduction) were still ongoing for a substantial proportion of participants. Thus, we expected a reduction in sick leave in the second year as compared with the pre-PSIW observation period.

### Measurement

#### Diagnoses

Psychiatric diagnoses were recorded by PSIW therapists according to the *International Classification of Diseases, 10th Revision* (*ICD-10*), chapter F.

#### Need for Treatment

During PSIW consultations, therapists assessed the need for treatment and made recommendations for treatment outside the PSIW when necessary. Altogether, 13 categories of recommendations were recorded. Up to three recommendations were made, for example, a first-line psychotherapeutic inpatient treatment followed by an outpatient treatment. To obtain a representation for the degree of need for treatment, we categorized recommendations into ordinal categories with ascending intensity of treatment: 1—PSIW currently sufficient, 2—treatment outside statutory health system (e.g., couple therapy, support groups), 3—outpatient mental health treatment (excluding psychotherapy), 4—outpatient psychotherapy, and 5—inpatient mental health treatment (psychiatric or psychotherapeutic hospital).

#### Use of Treatments in Regular Mental Health Care

Health insurance data provided information on inpatient treatments due to mental disorders, the duration (in days) as well as the main diagnoses (according to *ICD-10*, Chapter F). Participants were considered to have received outpatient psychotherapy when at least one billing number for a regular outpatient psychotherapy session (after up to 5 so-called “probatory” sessions) existed. Other specialized outpatient mental health care consultations were identified by the codes for specialist practitioners that included practitioners for psychiatry, psychotherapy, psychosomatic medicine, and neurology (the latter only when specific psychiatric billing numbers were present). A participant with at least one health insurance entry of those specialist practitioner codes was considered to have been treated in this sector.

#### Sick Leave

We aggregated medically certified sick-leave days irrespective of reason or diagnosis per quarter during the observation period of 1 year before and 2 years after the first PSIW consultation. In Germany, reports of medically certified sick leave to the insurance company are only mandatory when the sick leave period exceeds 3 days. Thus, we excluded all reports of 3 days or less because counting these reports is not considered reliable.

### Statistical Analysis

For the descriptive analysis, we described characteristics of the PSIW participants in terms of age, sex, number of PSIW consultations, diagnoses in PSIW, and recommendations for further treatment. Also, we recorded treatments preceding and following PSIW in regular health care settings. For preliminary analysis and descriptive information on the longitudinal development of sick leave, means (m), medians, and standard deviations (SD) of numbers of sick-leave days per quarter, half-years, and years were computed.

Sick-leave days were analyzed using a multilevel mixed-effects negative binomial regression (a generalized linear mixed model) for the following reasons: A multilevel mixed-effect model accounts for within-person correlations due to repeated observations by including random effects on the subject level and are therefore considered appropriate for longitudinal data structure (60). Count data follow characteristic distributions with positive skewness and are bounded by zero. If the variance is greater than the mean (overdispersion), negative binomial models are appropriate (61).

To specify parameters of the regression, we conducted preliminary analyses. As overdispersion was present in distributions of sick-leave days, we opted for a negative binomial regression. Further, we checked the model fits of the mixed-effects multilevel regression analysis compared with a simple negative binomial regression. That was tested with a likelihood ratio test for the null model and confirmed [chibar^2^ (01) = 127.83; prob > chibar^2^ = 0.001]. Also, we checked better fit of the negative binomial model compared with a Poisson model. This is indicated by an overdispersion in the dependent variable, reported in α > 0 (62). In the reported models, lnalpha is 1.45 and 1.46, respectively, corresponding to α [exp (1.45)] = 4.3, which supports the use of a negative binomial model. Furthermore, we used trajectory characteristics (see Section Results) and regression models using either years or half-years as units of observation to determine a reasonable observation unit for the analysis of sick-leave days in our main analysis and future studies. We considered that trajectories of half-years reveal important dynamics of change in sick-leave days, whereas quarters are too fine-grained, and years blur possible important dynamics. Also, regression models using half-years as observation unit of time showed better model fit than models using years (52). Consequently, we considered half-years to be an appropriate observation unit for sick-leave days.

For the regression models, outcome variable was the number of sick-leave days per quarter (level 1 variable), nested in participants (level 2 variable, random intercept). Independent variables were observation periods (half-years), gender, age, and indicators of impairment. In model 1, we examined the change in numbers of sick-leave days over time controlled for gender and age. In model 2, we also included variables indicating impairment: first, diagnosis of a mental disorder in the PSIW (yes/no), second, recommended treatment (PSIW sufficient, outpatient psychotherapy, inpatient mental health care, other recommendations); and third, mental health care (or diagnosis) before the PSIW (no mental health care before, mental health care by general practitioners or hospitals, mental health care by specialists).

The negative binomial regression coefficients were reported as incidence rate ratios (IRRs). An IRR indicates the ratio of increasing or decreasing numbers of sick-leave days compared with the respective baseline category. An IRR > 1.00, which is statistically significant, indicates an increasing trend, whereas an IRR < 1.00, a decreasing trend, respectively. Statistically significant differences were assessed at *p* < 0.05. Statistical analyses were performed using IBM SPSS v24 (63)/25 (64), STATA 15.1 SE (65), and Microsoft Office Excel 2007.

## Results

### Participant Characteristics and PSIW Usage

Of the 155 PSIW participants included, 84% (*n* = 130) were male. Mean age at first PSIW consultation was 45 (SD = 10.5) years. A total of 28% (*n* = 44) of PSIW clients had made use of specialized mental health care (inpatient or outpatient treatment) in the 12 months prior to first PSIW consultation. For another 34% (*n* = 52), we found one or more documented psychiatric diagnoses by general practitioners or disciplines other than specialized mental health care in the 12 months prior to PSIW. During PSIW sessions, 72% (*n* = 112) of participants received one or more psychiatric diagnoses according to *ICD-10*. Here, 12% (*n* = 19) were diagnosed with an adjustment disorder (F43.2X), and 60% (*n* = 93) were diagnosed with other (single or multiple) psychiatric disorder(s). Those were mainly affective and neurotic, stress-related, and somatoform disorders (44 and 42% of all diagnoses). Of those with psychiatric diagnoses, 64% had no contact to a mental health specialist in the year before. The number of consultations per participant ranged from 1 to 13, whereas the mean number of consultations in the PSIW was 4.1 (SD = 3.5, median = 2). The mean time span from first to last consultation was 80 days (SD = 115.2 days, median = 28 days).

### Recommended Treatments and Utilized Treatments

For 34% of PSIW participants (*n* = 53), PSIW consultations were considered as sufficient, and no further treatment was recommended. For another 33%, the recommended treatment was outpatient psychotherapy (*n* = 51). Twenty percent (*n* = 31) got a recommendation for inpatient mental health treatment (psychotherapeutic or psychiatric). Further treatments outside the statutory health system (e.g., counseling, self-help group) were recommended to 11% (*n* = 17). For three participants (2%), we had missing data due to incomplete records from the PSIW therapists. Recommendations for treatment in regular mental health care were given for 73% (*n* = 82) of the participants with mental illness(es) as diagnosed in PSIW.

In the 2 years following the first PSIW consultation, 48% (*n* = 74) of participants had no further treatment in regular health care. Some of those may have sought help in offers outside the regular health system (e.g., couples counseling), but our data set did not include information about this sector. Of all participants, 14% (*n* = 21) received outpatient psychotherapy, 17% (*n* = 26) had outpatient mental health treatment that did not include regular psychotherapy (e.g., psychiatric consultation and medication), and 19% (*n* = 29) received inpatient mental health treatment. An additional analysis of the number of days in inpatient mental health treatment (across all participants) showed a mean of 1.23 days (SD = 7.25 days) pre-PSIW, a mean of 8.75 days (SD = 21.78 days) in the first year post-PSIW, and a mean of 2.47 (SD = 13.82) in the second year post-PSIW. For five participants (3%), we could not specify the utilized treatments due to incomplete data.

The rate of participants who followed the given PSIW recommendations was 77% (*n* = 41) for “no further treatment needed” and 74% (*n* = 23) for “inpatient treatment.” In contrast, when outpatient psychotherapy was recommended, only 37% (*n* = 19) started such a therapy.

### Sick-Leave Days: Descriptive Results

On a descriptive level, data showed an increase of 5 sick-leave days from the year before (mean = 64.68, SE = 6.62, SD = 81.56, median = 31, and *n* = 152) to the first year post-PSIW (mean = 70.47, SE = 7.44, SD = 92.31, median = 33.5, and *n* = 154) and a reduction of 14 days from the year prior to PSIW to the second year after PSIW (mean = 50.97, SE = 6.54, SD = 80.14, median = 15, and *n* = 150). This change pattern could be observed for means and more clearly for medians. Table 1 shows sick-leave days in half-years and quarters. Using quarters as an observation unit gave more insight into the development of sick-leave days before and after PSIW start. There is an accelerating increase in sick-leave days from the first observed quarter (q −4) to the quarter just before the first PSIW session (q −1). This increase continues slightly in the first quarter after the start of PSIW (q +1). After that, sick-leave days drop, especially from second (q +2) to third quarter (q +3) after the first PSIW session. From q +4 to q +8, sick-leave days per quarter come to a relatively constant level of ~12–14 days, which is comparable to the pre-PSIW level in q −4 and q −3.

**Table 1:** Sick-leave days in half-years and quarters.

**Half-years**	**hy −2**		**hy −1**		**hy +1**		**hy +2**		**hj +3**		**hy +4**	
Mean	27.45		37.07		41.20		29.01		25.97		24.47	
SE	3.61		3.99		4.55		3.81		3.57		3.74	
SD	44.46		49.31		56.61		47.31		44.15		45.83	
Median	9		17		14		9		6		5	
*n*	152		153		155		154		153		150	
**Quarters**	**q −4**	**q −3**	**q −2**	**q −1**	**q +1**	**q +2**	**q +3**	**q +4**	**q +5**	**q +6**	**q +7**	**q +8**
Mean	13.57	14.37	16.61	20.46	21.30	19.90	15.65	13.36	14.04	11.99	12.77	11.62
SE	1.97	2.13	2.20	2.36	2.51	2.43	2.19	1.99	2.10	1.89	2.12	1.87
SD	24.27	26.30	27.17	29.14	31.27	30.25	27.12	24.67	26.05	23.39	26.01	22.91
Median	0	0	4	5	5	5	0.5	0	0	0	0	0
*n*	152	153	153	153	155	155	154	154	154	153	151	150

*PSIW starts at the beginning of year post 1, hy +1, and q +1, respectively (see also Figure 1). SE, standard error of the mean; SD, standard deviation*.

**Figure 2:**
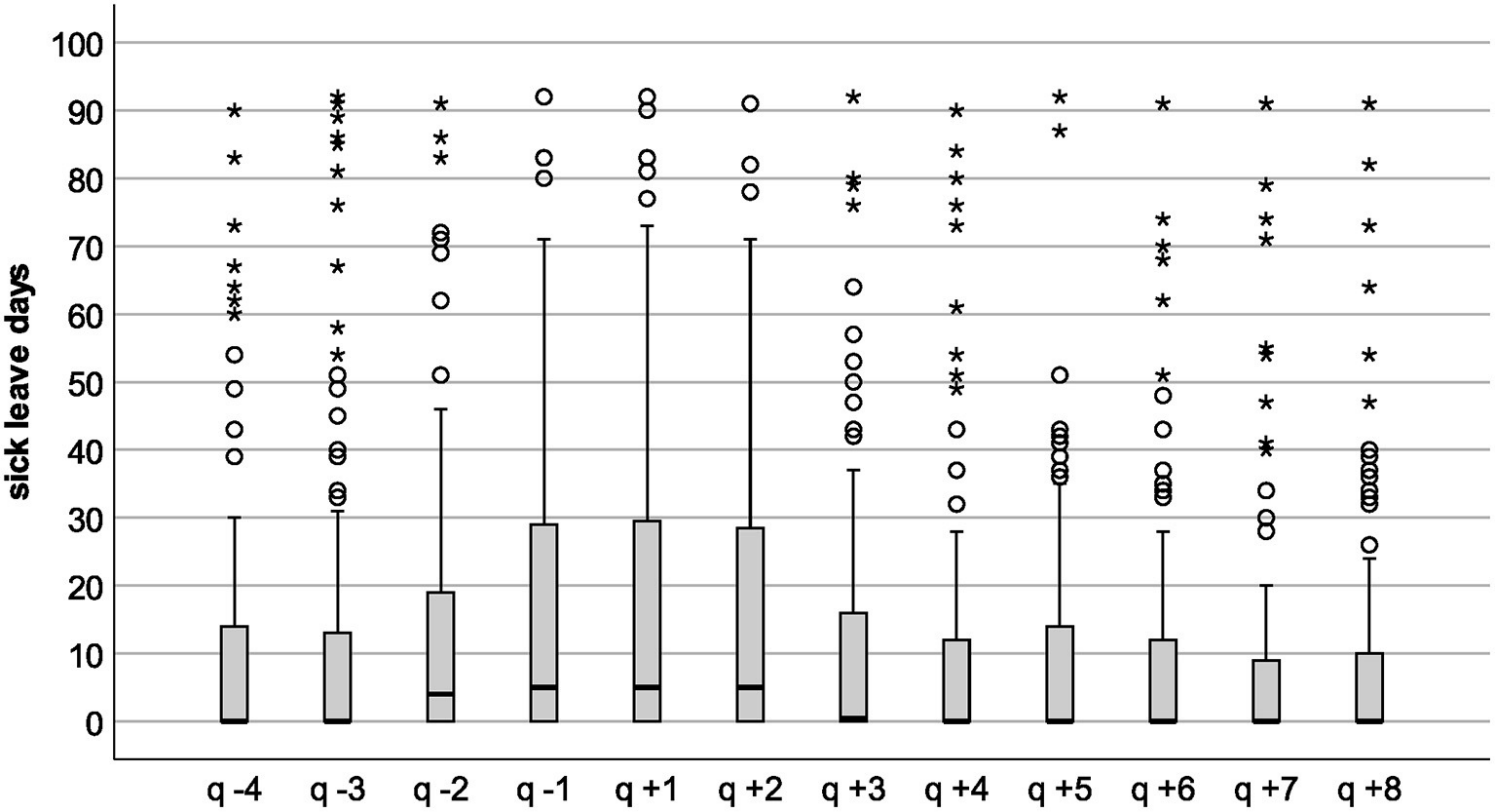
Boxplots of sick-leave days per half-year. hy, half-year, negative indexing marks the pre-observation period, positive indexing the post-observation period (Figure 1). The height of the box represents the interquartile range; the middle crossbar represents the median. Whiskers are 1.5 times the interquartile range; circles represent outliers with more than 1.5 times the interquartile range; asterisks represent extreme outliers with more than three times the interquartile range.

The distributions of sick-leave days (Figure 2) show a group of participants with a high number of sick-leave days, as well as a floor effect of participants with little or no sick-leave days. This is evident in pre-observation and post-observation periods.

### Sick-Leave Days: Results of Negative Binomial Regressions

Table 2 shows the results of the multilevel mixed-effects negative binomial regressions on sick-leave days in IRRs, the respective 95% confidence intervals, and *p*-values. The results of the multiple adjusted (age, sex) regression model 1 show a significant increase in the number of sick-leave days from half-year −2 to half-year −1 (baseline). In half-year +1, there is no change compared with baseline. Compared with baseline, a significantly reduced number of sick-leave days can be observed from half-year +2 through to half-year +4. There is no significant difference in sick-leave days between women and men as well as between age decades.

**Table 2:** Results of mixed-effects regression on sick-leave days.

	**Model 1**		**Model 2**	
	**IRR (95% CI)**	***p*** **Value**	**IRR (95% CI)**	***p*** **Value**
**Time (compared with half-year −1)**				
Half-year **–**2	0.67 (0.50–0.91)	0.011^*^	0.68 (0.49–0.93)	0.015^*^
Half-year +1	1.01 (0.75–1.36)	0.942	0.98 (0.72–1.32)	0.883
Half-year +2	0.63 (0.44–0.91)	0.014^*^	0.63 (0.44–0.91)	0.014^*^
Half-year +3	0.56 (0.38–0.81)	0.002^**^	0.58 (0.39–0.85)	0.005^**^
Half-year +4	0.57 (0.37–0.87)	0.010^*^	0.60 (0.39–0.94)	0.025^*^
**Female**	0.54 (0.26–1.12)	0.096	0.41 (0.21–0.81)	0.010^*^
**Age** (decade)^i^	1.26 (0.99–1.60)	0.063	1.15 (0.89–1.49)	0.279
**Health care pre-PSIW (compared with no)**				
Specialized mental health care			2.53 (1.24–5.16)	0.011^*^
Other			3.10 (1.77–5.42)	<0.001^***^
**Mental disorder diagnosed in PSIW (compared with no)**			0.74 (0.34–1.61)	0.448
**Recommended treatment (compared with no)**				
Offers outside health system			1.61 (0.69–3.76)	0.273
Outpatient psychotherapy			1.36 (0.67–2.74)	0.396
Inpatient mental health treatment			3.06 (1.55–6.06)	<0.001^***^
				
Constant	13.13 (9.61–17.63)	<0.001^***^	5.86 (2.98–11.53)	<0.001^***^
lnalpha	1.45 (1.29–1.61)		1.45 (1.29–1.61)	
ID (user) var (_cons)	1.94 (1.15–3.27)		1.38 (0.77–2.46)	
Log pseudolikelihood	−5,400		−5,383	

p Values indicate if the change in numbers is statistically significant (*p < 0.05, **p < 0.01, and ***p < 0.001).

*IRR, incidence rate ratio [the ratio of increasing (>1.00) or decreasing (<1.00) numbers of sick-leave days compared with the respective baseline category;*

i *decade is mean-centered (mean = 45); CI, confidence interval; ID (user) var (_cons), specifies the random intercept as a variance parameter on the individual user level*.

Model 2 is additionally controlled for indicators of impairment (diagnosed mental disorder in PSIW, recommended treatment, and mental health care before PSIW). It also shows a significant increase in the number of sick-leave days from half-year −2 to half-year −1 (baseline) and no change in half-year +1. Also, there is a significantly reduced number of sick-leave days from half-year +2 through to half-year +4 compared with baseline.

Model 2 yields the following results for the covariates of sick leave within the observed 3 years (all other predictors held constant, including time): Participants who had seen a mental health specialist prior to PSIW and those who had received a mental health diagnosis from other disciplines (other) showed significantly more sick-leave days compared with participants who had not been diagnosed and/or treated in the regular health system prior to PSIW. Those participants with at least one diagnosis of a mental illness during PSIW did not differ in the number of sick-leave days from participants without a diagnosis. When the recommendation for inpatient mental health treatment was given, participants had ~3 times as many sick-leave days than participants for whom PSIW was considered sufficient. In contrast, participants with a recommendation for outpatient psychotherapy or for help outside regular health care did not differ from those without further treatment recommendation.

### Exploration of Trajectories of Sick-Leave Days by Participant Subgroup

We also explored sick-leave days by indicators of impairment (treatment preceding PSIW, diagnosis of mental disorder in PSIW, and need for treatment assessed in PSIW) to identify differences in the time courses.

**Figure 3:**
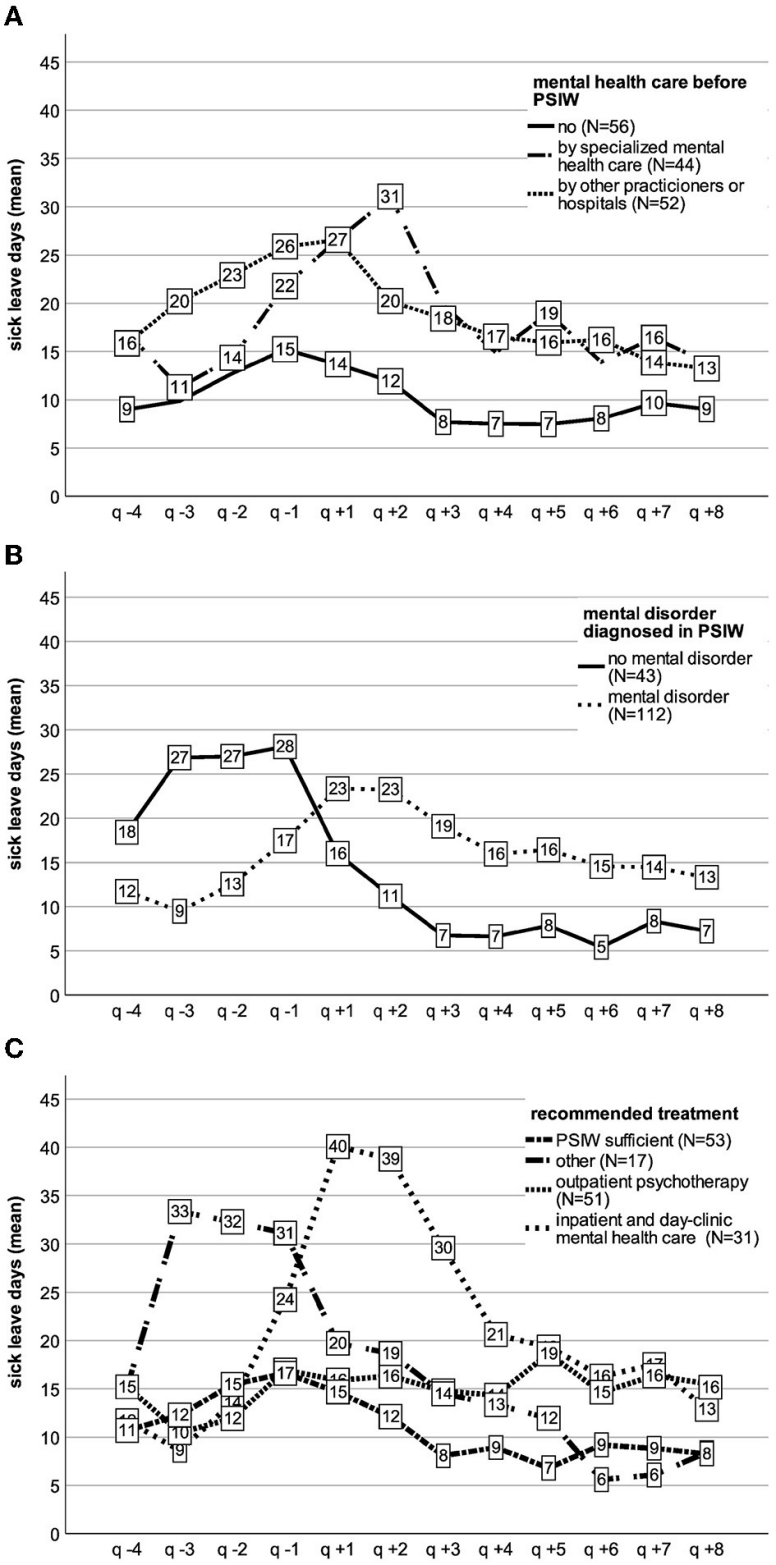
Sick-leave days over time by **(A)** health care preceding PSIW, **(B)** mental disorder, and **(C)** recommended treatment.

Figures 3A–C show the relation between those indicators of impairment and sick-leave days across the observed 12 quarters.

### Trajectories Over Time by Indicators of Impairment

Start of PSIW is at the beginning of q + 1. Negative indexing indicates quarters preceding first PSIW session, positive indexing quarters following first PSIW session (Figures 3A–C).

For participants without a previous record of mental illness or treatments, sick-leave days rose before PSIW start (q −4 to q −1) and then declined. A similar course with higher numbers can be observed for those with a diagnosed mental disorder from a non-specialized practitioner/hospital sick leave of participants who saw a mental health specialist prior to PSIW and show a different trajectory with a rise of sick-leave days that peaks in q +2 before declining (Figure 3A).

Also, trajectories varied between participants with and without diagnosis of a mental disorder in PSIW (Figure 3B). Sick-leave days for those with a mental disorder rose starting q −3 to q +1 and declined starting at q +3. For those without any mental disorder, sick-leave days showed a plateau before the first PSIW session followed by a decline afterwards. Further analysis for this group revealed a mean of 1.95 days (SD = 0.63 days) in hospital due to a somatic disorder in the year before PSIW start in comparison to a mean of 1.00 day (SD = 0.37) for all other participants. Also, 38% (*n* = 16) of this group were diagnosed with a mental disorder from regular health care in the year pre-PSIW.

Figure 3C shows that in case PSIW treatment was sufficient, mean numbers of sick-leave days rose before first PSIW session and declined after that. From q +3 on, numbers were lower than before PSIW. For participants with a recommendation for outpatient psychotherapy sick-leave days first declined from q −4 to q −3 and then also rose in q −1. After PSIW start, no substantial decline was observable. For participants with a recommendation for inpatient mental health care, a different trajectory with an incline from q −3 to q +1 and a decline starting q +2 could be observed. The small subgroup that received a recommendation for offers outside the regular health system showed a peak plateau of sick-leave days before PSIW, followed by a decline.

## Discussion

The aim of this study was to explore the characteristics of PSIW participants, their treatment histories, and the patterns of sick-leave days before and after PSIW. In addition, we tested the hypothesis that days of sick leave are reduced from the second year after the start of PSIW compared with before PSIW. This should provide information on participant subgroups, on the role of PSIW in the health care system, and on a possible effect of PSIW on sickness absence. Furthermore, indications for more specific research questions and designs should be derived.

During PSIW, 72% of the participants were diagnosed with at least one mental disorder. This is comparable to data from a similar offer of CMHW (41) and less than the available data for regular outpatient mental health care in Germany (66–68). Our analysis also showed that approximately two-thirds of participants had contact with a mental health specialist or were diagnosed with mental disorders(s) from other disciplines in the year preceding PSIW. For those without any previous record, PSIW seems to be the first place to seek help during their current mental health difficulties. A subgroup of those participants (17% of all) had only subsyndromal difficulties. This group seems to be reached at the most preferable time, which is before manifest illness. At this early stage, a short-term intervention can be sufficient to alleviate symptoms and to maintain or restore the ability to cope with everyday life and work. Although PSIW stand-alone interventions may not be adequate for all conditions, our insurance data-based analyses support previous findings (29, 42) that PSIW has the potential to reach employees at early stages of mental distress. In our sample, 84% of participants were male, which corresponds to the high number of male employees in the metal works sector and the investigated company (69). As men generally seek less treatment in regular mental health care settings (19, 25), our results also support previous findings that PSIW and related concepts (39, 41, 42) can facilitate access to adequate treatment for men. Concerning need for treatment, PSIW turned out as a sufficient treatment for approximately a third, whereas approximately half of the participants required more intensive therapies and were referred to regular mental health care. Comparison of PSIW pre-treatments to regular mental health care or other CMHW is difficult because of methodological differences and varying frames of reference (19, 42, 70). The same applies to referral rates, because those are dependent not only on the level of impairment but also on differing locally available treatment options [e.g., (41)].

Although PSIW can reach employees early in the development of a mental disorder, the larger subgroup of participants was diagnosed in PSIW with manifest illness(es). Approximately two-thirds of those had no contact to a mental health specialist in the year before. Those who did were still seeking help through PSIW, which implies that previous treatment was not sufficient and/or issues concerning workability was not resolved. Also, among the participants with manifest illness(es), most showed need for further treatment in regular mental health care. Thus, we conclude that PSIW is a flexible help offer in the workplace that can work as a stand-alone intervention but also seems to play an important role in “guiding” through the health care system. It can bridge a gap between distressed employees, regular mental health care, and workplace-centered interventions (e.g., assistance from company physician with workplace adjustments), which is often difficult to access in the regular system. We see this flexibility as a big advantage for employees and companies alike in addition to more structured workplace interventions that focus on specific subgroups.

Rates of adherence to PSIW recommendations were higher for inpatient treatment (74%) and lower for outpatient psychotherapy (37%). In our PSIW setting, inpatient treatment was an easily accessible option, as PSIW therapists were able to directly refer patients to the waiting list of a psychosomatic hospital. Previous research on recommendations for outpatient psychotherapy estimated adherence rates at 50–60%. Successful patients were found to be younger and often female and had higher depression severity (71). Starting psychotherapy also seems to depend on the level of hope, suffering, and initiative, while even a first session can lower the level of suffering (72). Lower adherence to the recommendation for outpatient psychotherapy might therefore reflect the high proportion of men, lower symptom severity, or lower sufferance due to one or more PSIW sessions. However, a similar PSIW concept with the possibility of a direct transfer to outpatient therapy showed adherence rates of 90% (41). Thus, difficulties to access outpatient psychotherapy in Germany (20, 21) also seem to play an important role. Besides possibilities of improving care either by even more efforts in supporting PSIW participants to start further treatment or by offering more sessions in PSIW, this points to the before stated necessity of change in the regular mental health care system to generally facilitate access for everybody (70, 73).

We found a substantial heterogeneity not only regarding participant characteristics and treatment histories, but also in regard to sick leave. PSIW participants showed a mean of 65 sick-leave days in the year before PSIW. In comparison, a mean of 16–18 sick-leave days per year for employees in the metal works sector (74) or 27–49 sick-leave days in the year before outpatient psychotherapy (54, 75) were reported. Although representative international samples found doubled days of sick leave or reduced workability in subjects with mental illness compared with those without (8, 76–79), the numbers in our sample seem high. A closer examination of the positively skewed distribution of sick-leave days preceding PSIW reveals that approximately one-third of the participants fell below the sector specific mean, while a smaller group of ~10% presented considerably high sick-leave days (>180 days/year), indicating long-term sick leave. The relatively high mean of sick-leave days in our sample might have occurred because of the specific setting, as well as access and referral practice in the context of the workplace. Accumulating sick leave may have increased the likelihood of visiting the occupational physician or social services (also through corporate return-to-work programs), who then recommended seeing a PSIW therapist. Also, elevated sick leave might be the consequence of methodological issues: as the present sample consists of the first PSIW participants after its implementation, employees with accumulated sick leave could have aggregated. The number of these participants might be lower in a longer-running PSIW, in which employees could potentially be reached at earlier stages in the development of their mental health difficulties.

Looking at the overall time course of sick-leave days, a rise in an exponential shape can be observed in the year before PSIW start. Similar data were reported before outpatient psychotherapy (75). In the half-year after the first PSIW session, numbers appear stable and then start to decline in the second half-year following the first PSIW session. Like the results on preceding treatments and diagnoses, this also implies that PSIW usage does not always happen at an early stage, but often only when the impairment is already high. For approximately half of PSIW participants, this resulted in need for further treatment in regular mental health care. Reducing symptoms and sickness absence of patients with manifest illness often takes time (58, 75), and reduction of sickness absence often only follows symptom reduction (36).

Regression analyses based on half-years showed a significant reduction of sick-leave days starting in the second half-year after the first session when compared with the half-year before PSIW start. This difference was found when controlled for age and sex or when indicators of impairment were additionally included as covariates. The observed reduction in sick leave is generally consistent with the reported reduction after outpatient psychotherapy (54, 57), inpatient psychosomatic rehabilitation in Germany (80), and other interventions that aim at reducing sickness absence (33). Regression analyses also showed significantly elevated sick-leave days across time for participants who received a recommendation for inpatient treatment (compared with PSIW sufficient) and for participants who had previously seen a mental health specialist or other practitioner due to mental health issues (compared with no previous contacts). There were no differences for age or psychiatric diagnoses across time, but women had less sick-leave days than men (only in model 2). It seems plausible that subjects who need inpatient treatment are generally more impaired and have more sick leave than outpatients. Also, previous contacts to the health system due to mental health issues imply more impairment and longer symptom duration. One might argue that because of the earlier onset of therapies before PSIW, those participants should present a lower level of sickness absence. However, on the basis of our results, it seems more likely that PSIW becomes an option of interest when previous therapeutic approaches were not sufficient.

Participant subgroups showed distinctive trajectories of sick-leave days. The trajectory of participants for whom PSIW was sufficient showed rising sick leave before and declining sick leave after the start of PSIW and is most indicative of a direct effect of PSIW on reducing sick-leave days. More difficult to interpret are the trajectories of those who received a recommendation for outpatient psychotherapy. They showed rising numbers of sick-leave days before PSIW, but no substantial decline afterward. This is likely due to the heterogeneity in the group in terms of strain, motivation, and treatment adherence (see above). Participants with referral to inpatient treatment showed an increase in sick leave starting in the quarter before and peaking in the two quarters after the start of the PSIW. This indicates a worsening of symptoms and reduced workability before PSIW, followed by sick leave due to inpatient treatment and return-to-work phase. Sick-leave days then continuously declined following q +3, which might reflect an ongoing process of restoring health and workability. The subgroup without a mental disorder (as diagnosed in PSIW) presented a high mean number of sick-leave days before PSIW followed by a decline after start. Prior to PSIW, they showed ~2 hospital days due to somatic illness (vs. 1 day for all other participants). In addition, 38% already had a diagnosis of a mental disorder prior to PSIW. As somatic and psychiatric comorbidity is associated with particularly high distress and high rates of sickness absence (81), and symptom reduction and capacity to work are not necessarily highly correlated (33, 34), this suggests that a part of this subgroup shows a combination of somatic illness (such as cancer diseases or injuries) and mental difficulties that lead to a complicated return to work even when manifest mental illness is not (or no longer) present. For these cases, PSIW may take tertiary preventive action and offer assistance in a more difficult return-to-work process.

Overall, the results support the assumption that PSIW can be beneficial in reducing sick-leave days across a highly heterogeneous group of participants. In addition, our results strongly suggest differential effects for subgroups of different indicators of impairment. As PSIW encompasses a very heterogeneous target group, our results seem to mirror the findings of international meta-analyses, which also report heterogeneous results and small effect sizes (33, 34).

Some limitations need to be considered. The lack of a control group does not allow any causal interpretations, so that differential effects of PSIW on sick-leave days cannot be determined. In the present study, it was not possible to utilize a control group due to company and data security interests. As stated, PSIW participants were very heterogeneous, which might have contributed to the unusual distributions and results. The distribution of sick-leave days as a count variable of the present highly heterogeneous sample leads to challenges in statistical modeling. Better fit of the used multilevel mixed-effects negative binomial regression with half-years as observation units of time compared with non-multilevel, Poisson models or using years as observation periods confirmed our choice (see above). Nonetheless, the effects of PSIW on sick-leave days are possibly overlaid by other interventions and may therefore be overestimated. To take this into account, assessment of interventions probably also needs to be more refined in terms of dosage and type. Other potentially influencing variables such as workplace conflicts have not been included. Also, representativeness and generalizability are limited because of the inclusion of only one company. As we excluded work disability periods of 3 days or less due to limited reliability, there might be an underestimation of overall work disability in the present sample.

Despite the aforementioned limitations, this study has several strengths. First, the continuous observation with data from a total of 3 years can be considered a strength, as standard pre–post studies under naturalistic conditions often exclude time periods during treatment (54–57). Second, the available data quality can be considered high as it is not biased by recall, motivation, or impairment of the participants (49, 50). Thus, the study concept allows a rather reliable picture of treatment courses and sick leave episodes of more than 3 days.

The results are of an exploratory character. However, they may be a useful contribution to the generation of more specific hypotheses as the data depict routine real-life treatment and sick leave courses. Further research on the role and effectiveness of PSIW as a promising concept of mental health care in the workplace should include control groups as well as dosage and type of treatment in the regular health system and PSIW (e.g., number of sessions, days in inpatient treatment or medication). This applies also to other potentially confounding variables such as severity of somatic illness or workplace conflicts. To make differential effects of PSIW visible, separate analysis of relevant participant subgroups (e.g., participants with subsyndromal distress, with apparent illness, or with return-to-work issues) is necessary.

To enhance the effects of the PSIW, efforts could be made to reach employees even earlier in the course of symptom development, for example, by increasing the familiarity for PSIW within companies and by working on organizational attitudes toward mental health issues. Although there is growing support for employee-centered interventions such as PSIW, more approaches are necessary. Sustainable mental health of employees is achievable only when organizations care for appropriate working conditions and support the return to work with adjusted workplaces (82, 83).

## Conclusion

Using routine data from PSIW and company health insurance, we found a broad spectrum of PSIW participants with regard to mental strain, preceding treatments, treatment needs, and sick leave. Besides employees with subsyndromal symptoms of psychosomatic distress, we also saw a subgroup with manifest illness in need for adequate treatment and/or with a substantial number of sick-leave days. This highlights the multiple roles of PSIW: besides adequate assessment and standalone short-term psychotherapy also guidance and facilitation regarding further treatments and support in a return-to-work process are important functions. Thus, PSIW works as a flexible collaborative care in the workplace. Compared with the half-year before PSIW, we found a reduction of sick leave beginning in the second half-year after PSIW start. This indicates a possible effect of PSIW on reducing sick leave and encourages future studies. Longitudinal courses of sick-leave days differ between the analyzed subgroups. Further research is needed to test causal effects and to identify specific effects of PSIW on sick leave for different subgroups. In addition to controlled designs, expanded participant characteristics and classification of intervention content are necessary to make differential effects visible.

## Data Availability Statement

The data analyzed in this study is subject to the following licenses/restrictions: the dataset for this study cannot be made publicly available due to data protection and legal reasons. Requests to access these datasets should be directed to jschneider@bkkvp.de.

## Ethics Statement

The studies involving human participants were reviewed and approved by Ethics Committee of the Ulm University Medical Centre, Ulm, Germany (04.05.2016; Ref No. 53/16). Written informed consent for participation was not required for this study in accordance with the national legislation and the institutional requirements.

## Author Contributions

MG, JW, MJ, and HG: conceptualization. MG and MJ: methodology. MG, JS, and SB: data acquisition and management. MG, MJ, and JW: data analysis and interpretation and second draft preparation. MG: original draft preparation. HG, JS, and SB: critically revision of the manuscript for important intellectual content. All authors reviewed and accepted the final form of the manuscript.

## Conflict of Interest

HG is director of a clinic and JW is head of a team of therapists that offers the service of PSIW to interested organizations and companies. MG was a therapist in a PSIW team. JS was chief executive and SB was deputy chief executive of a company health insurance that has offered PSIW to their members. The remaining author declares that the research was conducted in the absence of any commercial or financial relationships that could be construed as a potential conflict of interest.

## Publisher's Note

All claims expressed in this article are solely those of the authors and do not necessarily represent those of their affiliated organizations, or those of the publisher, the editors and the reviewers. Any product that may be evaluated in this article, or claim that may be made by its manufacturer, is not guaranteed or endorsed by the publisher.
